# Take control of expression: effector-mediated modulation of the host transcriptional machinery

**DOI:** 10.3389/fpls.2026.1771671

**Published:** 2026-01-27

**Authors:** Weiliang Zuo, Muye Xiao, Gunther Doehlemann

**Affiliations:** Institute for Plant Sciences and Cluster of Excellence on Plant Sciences (CEPLAS), University of Cologne, Cologne, Germany

**Keywords:** effector, gene expression, microbial virulence, plant immunity, transcriptional regulation

## Abstract

Interactions between plants and microbes that colonize them typically result in significant alterations of the host’s gene expression. Such transcriptional changes include modulation of immune responses, as well as orchestrating metabolic and developmental changes locally at sites of infection and systemically in the plant. Microbes colonizing diverse hosts have evolved cross-kingdom conserved mechanisms that utilize effectors to participate directly in host transcription process and actively rewrite its transcriptome for their own benefit. In this review, we highlight the mechanisms exploited by plant-colonizing microbes to manipulate the transcriptional machinery of their hosts, including interfering with and mimicking transcription factors and co-regulators. We provide a comprehensive overview of the functionalities of effectors beyond immune suppression and conclude that controlling the host transcriptome is crucial for establishing a favorable niche for microbial plant colonizers.

## 1 Introduction

The interaction between plants and microbes results in significant alterations to the host’s transcriptome. On the one hand, the perception of MAMPs (Microbe-Associated Molecular Patterns) and effectors from the microbes, or DAMPs (Damage-Associated Molecular Patterns) derived from plant itself can trigger PTI (Pattern-Triggered Immunity) and ETI (Effector-Triggered Immunity), and subsequent activation of the expression of immune related genes to limit microbial growth (Jones and Dangl, 2006; Bjornson et al., 2021; Ngou et al., 2021; Yuan et al., 2021). On the other hand, plant colonizing microbes actively modify the plants’ physiological processes by effectors to build a favorable niche for their proliferation (Gorshkov and Tsers, 2022). Therefore, in antagonistic interactions, i.e. plant-pathogen interactions, the outcome of the hosts’ transcriptome is the molecular tug-of-war of two competing processes. Conversely, in mutualistic interactions, plant gene expression needs to be balanced to allow successful establishment and to maintain symbiosis to the favor of both the host and its colonizer.

During host infection, microbes secrete so-called effectors that interact with host components to disarm the plant immune system and evade host recognition (Zhang et al., 2022), while also manipulating host metabolism and transcriptional programs for their benefit (Cai et al., 2023). The term “effector” is often used but not limited to proteins, since also non-proteinaceous molecules can perform “effector functions”, i.e. to modulate cellular functions that alter the biotic interaction.

The process of gene expression is initiated by the assembly of the core transcriptional machinery, which comprises RNA polymerase II and several general transcription factors (TFs) including TFIIA, TFIIB, TFIID, TFIIE, TFIIF and TFIIH, at the core promoter to form the preinitiation complex (Murakami et al., 2013). Gene-specific transcription factors bind to enhancers or other cis-regulatory elements and recruit transcriptional coregulators, including coactivators, corepressors, the *Mediator* complex, and the SAGA (Spt–Ada–Gcn5 acetyltransferase) complex. Together, these components determine tissue-, cell-, and stimulus-specific gene expression (De Mendoza and Sebé-Pedrós, 2019; Soffers and Workman, 2020; Chen et al., 2022; Blanc-Mathieu et al., 2024). Transcriptional coactivators and corepressors bind to the TFs and modulate their transcription activity. The mediator complex, on the other hand, bridges the gap between DNA-binding transcription factors and polymerase II (Pol II), as well as general TFs (Conaway and Conaway, 2011). The SAGA complex modifies and remodels the chromatin environment to facilitate transcription (Moraga and Aquea, 2015; Soffers and Workman, 2020). Subsequent to transcription, gene expression can be further fine-tuned through the activity of the RNA interference (RNAi) machinery, which is mediated by microRNAs (miRNAs) from endogenous non-coding RNA transcripts, or by small interfering RNAs (siRNAs) from double-stranded RNAs (dsRNAs) synthesised by endogenous RNA-dependent RNA polymerases, viruses or pathogens (Weiberg et al., 2013; Rosa et al., 2018; He et al., 2023). In addition, the epigenetic modification on the chromosome affects the accessibility of the DNA sequence to the transcriptional machinery, thereby defining the “on” and “off” states of the genes.

It has been demonstrated that effectors are capable of manipulating every aspect of host gene transcription. Microbes can directly control the host gene expression by the delivery of effectors that function as TFs to activate or suppress gene transcription, or they indirectly interfere with the host transcription machinery including TFs and coregulators, to rewrite the transcriptome. In addition, effectors can also affect the pre-transcriptional step by modifying the epigenetic state of host genome (Zhang and Cao, 2019) or post-transcription processes by hijacking the host RNAi machinery to adjust the mRNA levels (Wang et al., 2015; Ye and Ma, 2016).

In this article, we focus on effectors that influence transcription by manipulating components of the host transcriptional machinery, and highlight current mechanistic insights into how these effectors interfere with host transcription.

## 2 Transcription factor like effectors

### 2.1 Transcription activator-like effectors

Effector proteins can translocate into the host nucleus and function as TFs by directly binding to promoters of host genes and thereby modulating their expression. These effectors often contain characterized DNA-binding domains (DBDs) and a nuclear localization signal. This enables them to translocate from microbes into the nucleus of the host cell, where they bind to the promoter of host genes in a sequence-specific manner, thereby activating or suppressing their expression. A major research breakthrough was the identification of the “transcription activator-like effectors” (TALEs) (Boch et al., 2009). TAL effectors are characterized by the presence of a N-terminal type III secreted signal peptide, a central repeat region that determines a DBD, two nuclear localization signals, and a transcriptional activation domain (Boch et al., 2009; Teper et al., 2023). They are translocated to the host cell via the bacterial type-III secretion system, where they can bind directly to the effector binding elements (EBEs) within the promoter of host susceptible genes, and activate their expressions (Boch et al., 2009; De Lange et al., 2013). The binding specificity of TAL effectors is determined by the DNA binding domain, which is composed of near-identical tandem repeat arrays consisting of 33–35 amino acids. The 12th and 13th amino acid in the repeats are called repeat-variable di-residues (RVDs), which collectively determine the binding specificity to one single DNA base. The TALE repeats employ four RVDs (NN, NI, HD and NG) to recognize guanine, adenine, cytosine and thymidine, respectively, with some binding degeneracy within the code (Boch et al., 2009). *Ralstonia solanacearum* also encodes TAL effectors (RipTALs) (Li et al., 2013; Schandry et al., 2016). However, they exhibit divergent preferences in the 5’ terminal of the EBE sequences. *Ralstonia* TAL effectors prefer the 5’ guanine while *Xanthomonas* TAL effector prefer the 5’ thymine (De Lange et al., 2013).

**Figure 1:**
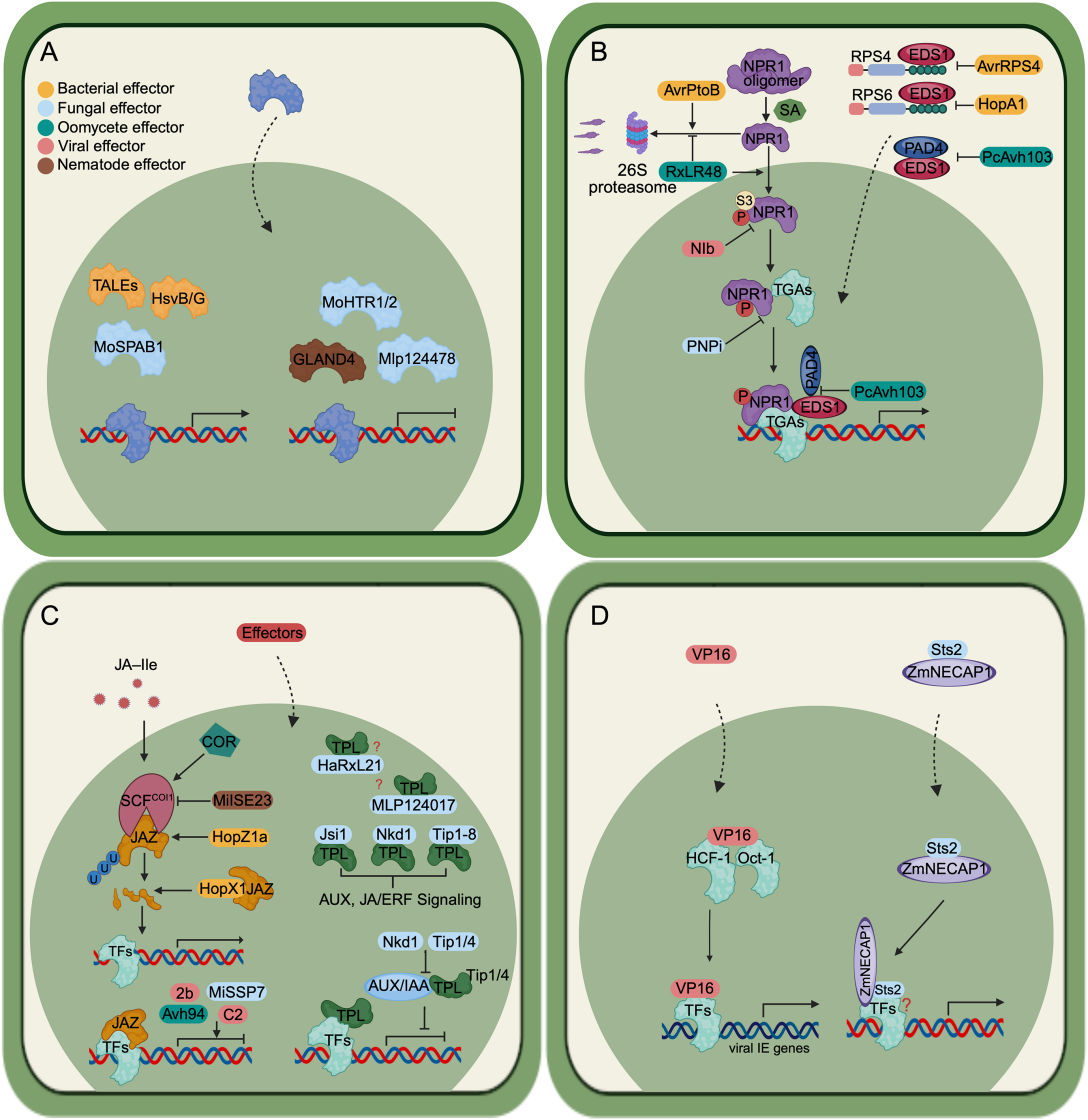
Effector strategies targeting host transcription in plant-colonizing microbes. **(A)** Nuclear-localized effectors from bacteria and fungi can function as transcription factors or transcription factor–like proteins that directly bind host DNA to activate or repress specific host genes. Examples include bacterial type III–secreted TAL (transcription activator–like) effectors from *Xanthomonas* and *Ralstonia*, HsvB *and* HsvG from *Pantoea agglomerans*, and MoSPAB1 from *Magnaporthe oryzae*, which activate host gene expression whereas MoHTR1 and 2 from *M. oryzae*, Mlp124478 *from Melampsora larici-populina and* GLAND4 from nematodes, which suppress host gene expression. **(B)** Effectors interfere with immune-related transcription by targeting transcriptional coactivators and upstream immune signaling components. During infection, the coactivators NPR1 (Nonexpressor of Pathogenesis-Related Genes 1) and EDS1 (Enhanced Disease Susceptibility 1) translocate to the nucleus to activate salicylic acid (SA)–responsive resistance genes. AvrPtoB, which is found in *Pseudomonas syringae*, mediates the degradation of NPR1. In contrast, RxLR48, which is found in *Phytophthora capsici*, prevents NPR1 degradation. PNPi from *Puccinia striiformis* f. sp. *tritici* competes with the transcription factor TGA2.2 for interaction with NPR1. Nib from the turnip mosaic virus inhibits the post-translational modification of NPR1. AvrRPS4 and HopA1 from *P. syringae* interact with EDS1, disrupting its interaction with the R proteins RPS4 and RPS6. In contrast, *P. capsici* PcAvh103 disrupts the interaction between EDS1 and PAD4. **(C)** Effectors reprogram phytohormone-responsive transcription by targeting transcriptional corepressors and hormone signaling pathways. JAZ (jasmonate ZIM-domain) proteins regulate jasmonic acid (JA)–dependent defense, while TOPLESS/TOPLESS-RELATED (TPL/TPR) corepressors control diverse phytohormone pathways, including auxin signaling. Coronatine (COR) is a phytotoxin that mimics JA-IIe (jasmonoyl-L-isoleucine), activating the host JAZ degradation pathway. HopX1 and HopZ1a, which are found in*P. syringae* function as *a* cysteine protease and an acetyltransferase, respectively, to facilitate JAZs degradation. Conversely, Avh94 from *P, sojae* MiSSP7 from *Laccaria bicolor*, MiISE23 from *Meloidogyne incognita*, the 2b protein from cucumber mosaic virus, and C2 proteins from geminiviruses stabilize JAZ proteins. The *Melampsora laricipopulina* effector MLP124017, the *Hyaloperonospora arabidopsidis* effector HaRxL21 and 10 effectors from *Ustilago maydis* interact with TPL/TPR corepressors to modulate diverse hormone signaling pathways. By manipulating JAZs, TPL/TPRs, SCF complexes and associated transcription factors, the effectors can shift the expression of genes regulated by hormones to favour microbial colonisation. **(D)** Effectors can function as transcriptional coregulators that redirect host transcriptional machinery. The viral effector VP16 from Herpes simplex virus interacts with the host transcription factors Oct-1 and HCF-1 to activate viral immediate-early gene expression. The *Ustilago maydis* effector Sts2 acts as a transcriptional (co)activator (TAE). It interacts with the maize coactivator ZmNECAP1 and promoting expression of maize leaf developmental regulators. The figure is created via biorender.

The first described TAL effector, AvrBs3, was identified in *Xanthomonas campestris* pv. *vesicatoria*. AvrBs3 activates UPAs (up-regulated by AvrBs3) gene expression. In pepper, AvrBs3 induces the expression of the TF UPA20, resulting in an increase in the host cell size to form hypertrophic mesophyll cells (Kay et al., 2007). Another common TAL effector targets are *SWEET* (Sugar Will Eventually Be Exported Transporter) genes, which encode sugar transporter proteins (Yang et al., 2006; Antony et al., 2010; Chen et al., 2010; Yu et al., 2011; Cohn et al., 2014; Zhou et al., 2015; Cox et al., 2017; Xu et al., 2024; Charleux et al., 2025; Khadgi et al., 2025). *Xanthomonas* species infecting different hosts process different TAL effectors to activate the expression of diverse *SWEET* genes, suggesting that this function is an evolutionary conserved mechanism adopted by *Xanthomonas* species. In addition, TAL effectors can activate the expression of non*-SWEET* gene including UPAs, TFs and splicing regulators (Kay et al., 2007, 2009; Sugio et al., 2007; Cai et al., 2017; Chen et al., 2024, 2025), and a *Ralstonia* TAL effector Brg11 activates the expression of arginine decarboxylase (ADC) genes (Gallas et al., 2024). Interestingly, truncated TAL effectors have been identified in the symbiotic bacterium *Mycetohabitans* (formerly *Burkholderia*) *rhizoxinica*, which infects the fungus *Rhizopus microspores*. The colonization of *Mycetohabitans* is a crucial factor for Rhizopus sporulation (Partida-Martinez et al., 2007; Richter et al., 2023). Despite the absence of type III secreted signal peptide, nuclear localization signal and activation domain, *Mycetohabitans* TAL effectors have been shown to translocate into the nucleus of the host cell via an as yet undetermined mechanism (Carter et al., 2020). *Mycetohabitans* TAL effectors have diverse functions. The deletion of Btl19–13 did not inhibit bacterium infection, however, the infected fungal cells show reduced tolerance to membrane stress (Carter et al., 2020). MTAL1–3 are *Mycetohabitans* factors important for the fungal host to control the sporulation (Carter et al., 2020), and MTAL1 protect bacterium from trapping and improve its survival within the fungal hyphae (Richter et al., 2023). In summary, TALEs are evolutionarily conserved at the structural and mechanistic level and can be found in diverse bacterial interactions, but their DNA-binding specificities are highly diversified and rapidly evolving (**Figure 1A**).

### 2.2 Beyond TALEs: other transcription factor-like effectors

In addition to the well-studied TAL effectors, TF-like effectors with other DBDs have been discovered. The epiphyte and commensal bacterium *Pantoea agglomerans* has evolved the ability to induce gall formation on specific hosts. *P. agglomerans* pv. *gypsophilae* and *P. agglomerans* pv. *betae* cause galls on gypsophila, whereas only *P. agglomerans* pv. *betae* can do so on beets (Barash and Manulis-Sasson, 2009). The plasmid-encoded type III effector HsvG determines the host specificity of both pathovars on gypsophila, while its homolog HsvB determines the host specificity on beets (Barash and Manulis-Sasson, 2007). Interestingly, HsvG and HsvB contain two nuclear localization signals (Weinthal et al., 2011) and five helix-turn-helix (HTH) motifs that constitute a putative DBD and function as transcriptional activators (Nissan et al., 2006). HsvG binds directly to the promoter of *HSVGT*, a putative TF gene in *Gypsophila paniculate*, and activates its expression (Nissan et al., 2012). In contrast, the target of HsvB remains unknown.

The fungal rice blast pathogen *Magnaporthe oryzae* encodes several TF-like effectors. One example is the effector MoSPAB1, which functions as a transcription activator. During infection, MoSPAB1 is translocated to the host nucleus and competes with the host transcriptional repressor MYBS1 to bind to the promoter of *Bsr-d1*, a C2H2-type TF regulating the expression of peroxidase genes (Weinthal et al., 2011). MoSPAB1 activates its expression, which in turn leads to the degradation of H_2_O_2_ produced in the PTI response (Li et al., 2017; Zhu et al., 2023). Also the homologs of MoSPAB1 in the anthracnose fungi *Colletotrichum fructicola* and *Colletotrichum sublineola* activate *Bsr-d1* expression in kiwifruit and sorghum, respectively, indicating a conserved virulence mechanism exploited by fungal pathogens (Zhu et al., 2023). In addition, *M. oryzae* encodes two transcription repressor effectors, MoHTR1 and MoHTR2 (*M. oryzae* Host Transcription Reprogramming 1 and 2). Both effectors are secreted via the biotrophic interfacial complex (BIC) and translocate into host nuclei. MoHTR1 is SUMOylated to enhance its stability and interaction with rice importin α, facilitating its translocation (Kim et al., 2020). Both MoHTR1 and MoHTR2 directly bind to the EBEs (CAATCTTC for MoHTR1) and (CCACCTCC for MoHTR2) in the promoters of rice genes, thereby suppressing their expression, despite the absence of intrinsic repressor activity for these effectors (Kim et al., 2020). Similarly, the basidiomycete rust fungus *Melampsora larici-populina* encodes the effector Mlp124478, which contains a putative nuclear localization signal and DBD, and may associate with the TGA1a-binding sequence in host DNA (Ahmed et al., 2018). The plant-parasitic cyst nematodes *Heterodera glycines* and *Heterodera. schachtii*, which infect soybean and sugar beet, respectively, secrete the effector GLAND4 into the plant nucleus. GLAND4 functions as a transcriptional suppressor that inhibits the expression of two lipid transfer protein (LTP) genes, thereby increasing host susceptibility to *Pseudomonas syringae* (Barnes et al., 2018).

Thus, effector that function as transcription factors allow microbes to control the expression of host genes with EBEs in their promoters with just one effector protein. Although the impact may be limited to a small number of host genes, these genes are often critical for the microbes’ virulence and benefit.

## 3 Effectors interfering with transcription factors/cofactors

The transcriptional modulation of host cells can also be achieved by effector proteins that target components of the host transcription machinery. This impedes their activities during infection, thereby manipulating host gene expression. In a recent review, Xiang et al. (2025) highlighted the current knowledge on effectors that target host TFs to modulate their function. Here, we focus on effectors that interfere with transcriptional coregulators, which include transcriptional coactivators, transcriptional co-suppressors and mediators (**Figures 1B, C**). Transcriptional coactivators typically remain in an inactive state in the host cytoplasm under normal condition. During defense responses, they are activated and translocated to the nucleus, where they activate the expression of immune responsive genes (Khan et al., 2022). In contrast, transcriptional co-suppressors bind to host TFs to inhibit the activation of downstream genes. Under pathogen attack, transcriptional co-suppressors are degraded to release the TFs to activate their target genes. Mediator interacts with TFs and transcriptional coregulators to assemble the transcriptional machinery to initiate transcription (Moore et al., 2011).

### 3.1 Targeting the transcriptional coactivator networks: NPR1 & EDS1

A central regulator of plant immunity is the transcription coactivator NPR1 (Non-Expressor of Pathogenesis-Related Genes 1), which has been identified through genetic screens with *Arabidopsis thaliana* mutants that were defective in the induction of systemic acquired resistance in response to the defense-inducing phytohormone salicylic acid (SA) (Cao et al., 1994). NPR1 contains an N-terminal BTB/POZ (Broad-complex, Tramtrack, Bric-à-brac) domain, a central ankyrin-repeat domain, and a C-terminal transactivation domain. Importantly, NPR1 has been identified as a receptor for salicylic acid (SA) (Wu et al., 2017). In non-challenged cells, NPR1 is maintained in the cytoplasm as an inactive oligomer through redox-dependent disulfide bonds. Upon SA accumulation and a shift to a more reducing cellular redox state, NPR1 is reduced to monomers, which bind SA (Mou et al., 2003). The monomeric NPR1 then undergoes multiple post-translational modifications and translocates into the nucleus, where it interacts with TGA transcription factor dimers to form an enhanceosome that activates PR gene expression (Kumar et al., 2022).

Another transcriptional coactivator, which plays a central role in plant SA-dependent defense and TIR-NB-LRR (Toll-Interleukin-1 receptor-Nucleotide Binding-Leucine Rich Repeat) receptor-mediated disease resistance is EDS1 (Enhanced Disease Susceptibility1) (Falk et al., 1999; Lapin et al., 2020).

EDS1 and its two sequence-related interactors, PAD4 (phytoalexin deficient 4) and SAG101 (senescence-associated gene 101), belong to a plant-specific protein family characterized by an N-terminal α/β hydrolase (lipase-like) domain and a highly conserved C-terminal α-helical bundle, the EDS1–PAD4 (EP) domain (Lapin et al., 2020). Different EDS1 complexes lead to distinct modes of immune regulation (Wagner et al., 2013). The EDS1–PAD4 nucleocytoplasmic complex reinforces basal immunity, which is partly mediated by salicylic acid (SA) signaling (Feys et al., 2001), whereas the EDS1–SAG101 nuclear complex promotes effector-triggered immunity (ETI) downstream of TIR-NLR proteins (Feys et al., 2005). Later, EDS1 was shown to contain acidic transactivation domains and to reprogram the host transcriptome by interacting with CDK8 (cyclin-dependent kinase 8) as part of the Mediator complex. Furthermore, NPR1 and EDS1 cooperate during SA signaling. NPR1 recruits EDS1 to the PR1 promoter to enhance its expression, while NPR1 upregulates EDS1 transcription and EDS1 stabilizes NPR1 during the plant immune response (Chen et al., 2021).

Given the critical role of NPR1 in SA-mediated immunity, research has focused on identifying pathogen effectors that interact with NPR1 and subvert SA-dependent defense. AvrPtoB, a type III effector of *Pseudomonas syringae*, contains an N-terminal Pto-interacting domain (PID) and a C-terminal U-box type E3 ubiquitin ligase domain (Xiao et al., 2007). In the presence of SA, AvrPtoB strongly interacts with NPR1 and mediates its degradation via the host 26S proteasome, a process dependent on its E3 ligase activity (Chen et al., 2017). A yeast two-hybrid screen using wheat NPR1 as bait identified the effector PNPi (Puccinia NPR1 interactor) from *Puccinia striiformis* f. sp. *tritici* as a nuclear interactor of wheat NPR1. PNPi binds NPR1 through its C-terminal DPBB_1 (double-psi beta-barrel) domain and competes with the transcription factor TGA2.2 for NPR1 interaction (Wang et al., 2016). Similarly, the RxLR effector RxLR48 from the oomycete pathogen *Phytophthora capsici* was found to interact with NPR1. In contrast to *P. syringae* AvrPtoB, RxLR48 promotes NPR1 nuclear localization and accumulation, preventing its degradation via the 26S proteasome and thereby modulating NPR1 turnover to suppress plant immunity (Li et al., 2019). Also viruses can target NPR1 to facilitate infection. Turnip mosaic virus encodes an RNA-dependent RNA polymerase, NIb (NUCLEAR INCLUSION B), which binds NPR1 at its SUMO-interacting motif 3 exclusively in the nucleus, preventing its sumoylation and phosphorylation and thereby disrupting NPR1-dependent immune responses (Liu et al., 2023).

Compared to NPR1, EDS1 is less frequently reported as an effector target. However, the *P. syringae* effectors AvrRPS4 and HopA1 interact with EDS1, disrupting the EDS1–RPS4 and EDS1–RPS6 complexes at the cytoplasmic membrane, respectively, thereby activating RPS4- and RPS6-mediated immunity to restrict bacterial growth (Bhattacharjee et al., 2011; Heidrich et al., 2011). Another effector targeting EDS1 comes from *Phytophthora capsici*: PcAvh103 is required for infection and binds the lipase domain of EDS1, disrupting its interaction with PAD4 and suppressing plant defense (Li et al., 2020). Together, NPR1 and EDS1 as central hubs of plant SA-mediated immunity are targeted by diverse pathogen effectors to modulate immune outcomes (**Figure 1B**).

### 3.2 Modulation of transcriptional repressors: JAZ

JAZ (jasmonate ZIM-domain) proteins are a class of transcriptional repressors that negatively regulate JA (jasmonic acid)-related defense and growth (Liu et al., 2021; Zhao et al., 2024). JAZ proteins contain three distinct domains. The N-terminal NT domain interacts with DELLA protein to inhibit the JA signaling pathway (Hou et al., 2010). The central ZIM domain contains a 28 amino acids TIF [F/Y] XG sequence, which mediates the interaction with NINJA (Novel Interactor of JAZ) (Pauwels et al., 2010). The C-terminal Jas domain is characterized by the core SLX2FX2KRX2RX5PY sequence, which is essential for JAZs function by binding to different regulatory proteins (Melotto et al., 2008). In the absence of JA, JAZs interact and block the downstream transcription factor MYC3/4 from activating their target genes by recruiting the general corepressors TOPLESS (TPL) and TPL-Related proteins through an interaction with NINJA (Pauwels et al., 2010). While bioactive JA is perceived, JAZs are targeted by the COI1 (CORONATINE INSENSITIVE 1) protein, a part of the SCF (Skip/Cullin/F-box)-COI1 E3 ubiquitin ligase complex, for degradation, which releases the inhibited TFs and activates JA responsive genes (Thines et al., 2007; Pauwels and Goossens, 2011). Several plant pathogens were found to deploy effectors to manipulate JAZ proteins, thereby either activating or suppressing JA signaling. One classic example is the non-proteinaceous effector coronatine (COR) produced by *P. syringae*. COR is a phytotoxin mimic of JA-IIe (jasmonoyl-L-isoleucine, the active signal in the JA signaling pathway) and it is 1,000-fold more active than JA-IIA in promoting the interaction of JAZ and COI1 (Katsir et al., 2008). In addition, *P. syringae* encodes several type III effector proteins, which interfere JAZ proteins through similar mechanisms. HopX1 from *P. syringae pv. tabaci* (*Pta*) is a cysteine protease that interacts with and directly degrades the JAZ5 protein (Gimenez-Ibanez et al., 2014), whereas HopZ1a has acetyltransferase activity and acetylates JAZ proteins to promote their degradation (Jiang et al., 2013). Conversely, effectors can also interact with JAZ proteins to stabilize them. For example, the RXLR effector Avh94 from *Phytophthora sojae* interacts with soybean JAZ1/2 proteins (Zhao et al., 2022), and MiSSP7, secreted by the mycorrhiza fungus *Laccaria bicolor*, interacts with Populus JAZ6 (Plett et al., 2014); both effectors stabilize their respective JAZ targets. Similarly, MiISE23 from the plant-parasitic nematode *Meloidogyne incognita* competes with COI1 for binding to JAZ proteins, thereby preventing their degradation during JA response (Shi et al., 2025). Furthermore, 2b protein from cucumber mosaic virus, and C2 proteins from geminiviruses were also found to interact with and stabilize JAZ proteins to suppress JA-related repones (Wu et al., 2017; Rosas-Diaz et al., 2023). Thus, JAZ proteins as regulators of jasmonate signaling are targeted by diverse effectors to either activate or suppress JA-mediated defenses (**Figure 1C**).

### 3.3 TOPLESS proteins as effector target hubs

Another central hub that regulates plant gene expression is the TOPLESS/TOPLESS-RELATED (TPL/TPR) protein family, which belong to the Groucho (Gro)/Tup1 family. TPL proteins function as transcriptional corepressors that regulate a wide range of plant processes, including nearly all hormone signaling pathways, root and leaf development, reproduction, and responses to both abiotic and biotic stresses (Plant et al., 2021; Saini and Nandi, 2022). TPL proteins contain a N-terminal TPD (TOPLESS domain) domain, which consists of a Lissencephaly Homologue (LisH) domain, a C-terminal to LisH (CTLH), and a CT11-RanBPM (CRA) domain. The TPD interacts with transcription factors containing repression domain (RD) sequences, including the EAR domain—defined as (L/F)DLN(L/F)xP, encompassing LxLxL, DLNxP, and DLNxxP motifs, and also including FDLNI—as well as the (R/K)LFGV and TLxLF sequences (Causier et al., 2012). The C-terminus of TPL proteins contains WD40 repeats, which provide a scaffold for protein–protein interactions (Collins et al., 2019).

Recent work has shown that TPL proteins are common targets of microbial pathogens (**Figure 1C**). Typically, effector proteins targeting TPLs carry an EAR domain, which can interact with TPL/TPR proteins to modulate host hormone signaling during infection (Khan and Djamei, 2024). A screen of 20 effectors from the poplar leaf rust pathogen *Melampsora laricipopulina* in *Nicotiana benthamiana* for their subcellular localization and plant interactors revealed that MLP124017 interacts with the TPL protein NbTPR4 (Petre et al., 2015). HaRxL21, an RxLR effector that is conserved in multiple *Hpa* isolates, interacts with *Arabidopsis* TPL and TPL-related 1 proteins through its EAR motif, which is essential for the effector’s virulence function. Overexpression of HaRxL21 in plants can suppress plant immunity to both biotrophic and necrotrophic pathogens (Harvey et al., 2020). Notably, in *Ustilago maydis*, a total of 10 effectors have been identified that interact with TPL/TPR proteins. The effector Jsi1 (jasmonate/ethylene signaling inducer 1) contains the DLNxxP motif, which interacts with the second WD40 domain of maize TPL1 and Arabidopsis TPL/TPR proteins. Although deletion of *jsi1* does not affect *U. maydis* virulence, its overexpression in *Arabidopsis* activates the ethylene response factor (ERF) branch of the jasmonate/ethylene (JA/ET) signaling pathway (Darino et al., 2021). Nkd1 (Naked1) was first identified as a PTI inhibitor through a screen of *U. maydis* effectors for their ability to suppress PAMP-triggered ROS burst. Nkd1 binds to TPL via its EAR motif, thereby preventing the recruitment of ZmIAA5 to the TPL/TPR proteins to de-suppress the auxin and jasmonate signaling (Navarrete et al., 2022). In addition, five of the eight effectors located in the *U. maydis* effector gene cluster 6A region (Kämper et al., 2006) were shown to interact with TPL proteins. These effectors have been identified to activate auxin-responsive gene expression. Two of these effectors were found to compete with ZmIAA3 and ZmIAA8 for TPL interactions. However, only the deletion of the whole cluster resulted in reduced virulence, suggesting their functional redundancy (Bindics et al., 2022). A further screen of 297 *U. maydis* effector candidates discovered three additional effectors that interact with TPL, including the previously identified organ-specific effector Tip6 (Schilling et al., 2014; Huang et al., 2024; Khan et al., 2024). Tip6 interacts with the N-terminal of TPL protein with its two EAR motifs, altering its nuclear localization and thereby disrupting the regulation of AP2/ERF B1 subfamily transcription factors (Huang et al., 2024). Although all of these effectors interact with TPL proteins, not all produce detectable virulence phenotypes upon gene deletion, suggesting a complex, spatiotemporal network of TPL-interacting effectors and their host targets in *U. maydis*.

### 3.4 Mediator, the SAGA complex and more

While the above given examples of plant hubs in the regulation of immune-related gene expression have received considerable attention in recent literature, effector targets are clearly not limited to these cases. For instance, effectors have also been found to target the *Mediator* complex to disrupt host gene transcription. *Mediator* is a large multisubunit complex that bridges transcriptional regulators at enhancers or other cis-regulatory sequences with RNA polymerase II at the transcription start site (Conaway and Conaway, 2011; Chen et al., 2022). The oomycete *Hpa* translocates the effector HaRxL44 into the host nucleus, where it interacts with the *Mediator* subunit MED19a, leading to its degradation and thereby attenuating SA-responsive defense (Caillaud et al., 2013).

The SAGA complex generally functions as a transcriptional coactivator that modulates transcription. It consists of more than 20 subunits, which are grouped into four functionally independent modules: the deubiquitinating (DUB) module; the histone acetyltransferase (HAT) module; the core module; and the TBP-associated factor (TAF) module (Moraga and Aquea, 2015; Soffers and Workman, 2020). General Control Non-depressor 5 (GCN5) and Alteration/Deficiency in Activation 2 (ADA2) are two key subunits of the HAT module. The pathogen *P. sojae* produces the effector PsAvh23, which interacts with the host ADA2 protein, disrupting the formation of the ADA2-GCN5 sub complex and leading to reduced levels of H3K9 acetylation and repressed defense gene expression (Kong et al., 2017).

In summary, it is evident that transcriptional coregulators play a key role in immune responses associated with phytohormones. Plants depend on these coregulators to detect fluctuations in hormone levels, enabling them to respond rapidly to attacks from pathogens by shifting from a standby state to a state of immunity. Interfering with plant transcriptional coregulators therefore evolved as a widely conserved and effective strategy employed by microbes colonizing plants from different kingdoms to manipulate host gene expression for their own benefit (**Figure 1C**).

## 4 Effectors acting as transcriptional coregulators

As outlined above, effectors can function as transcription factor by directly binding to the promoter of host genes. More recent evidence shows that effectors can also act as transcriptional coregulators, to participate or hijack the host transcriptional machinery. An example found in a mammal virus is the VP16 protein from Herpes Simplex virus 1 (HSV-1). VP16 functions as transactivator to activate the expression of viral immediate-early genes (Fan et al., 2020). During infection, VP16 interacts with the host transcription factors Oct-1 (Octamer-binding protein 1) and HCF-1 (host cell factor 1), and recruits a series of transcriptional coregulators to activate transcription (Babb et al., 2001; Herrera and Triezenberg, 2004; Vojnic et al., 2011). In plant-microbe interactions, the *U. maydis* effector Sts2 (small tumor on seedlings 2) has been identified as a transcriptional activator effector (TAE) (Zuo et al., 2023). Sts2 is an organ-specific (Skibbe et al., 2010; Schilling et al., 2014) and cell type-specific effector (Matei et al., 2018), whose virulence function is involved in the hyperplasic tumor formation in maize leaves (Zuo et al., 2023). Sts2 orthologs from *U. maydis* and its close pathogenic relative *Sporisorium reilianum* are differentially regulated during infection and, moreover, the orthologous proteins have different virulent functions (Zuo et al., 2021). Sts2 translocates into the host nucleus during infection and functions as transcription (co)activator to activate the expression of leaf developmental regulators. Interestingly, *U. maydis* Sts2 interacts with maize NECAP1, a protein that is also a functional transcription activator (Zuo et al., 2023). However, so far it is unclear if and how the interaction with maize NECAP1 is required for Sts2 function.

While Sts2 contains an acidic transactivation domain, no typical nuclear localization signal or DBD can be identified, which implies that it might need to recruit a host TFs or DNA binding proteins, although further evidence is required (Zuo et al., 2023).

The discovery of effectors that function as transcriptional coregulators suggests a novel virulence mechanism, in which pathogens hijack host transcription factors or DNA-binding proteins to enhance expression of their target genes in support of pathogenesis (**Figure 1D**).

## 5 Conclusions and perspective

Plant colonizers from diverse kingdoms secrete effectors that can function as TFs or transcriptional coregulators, in addition to effectors that interfere with host TFs and coregulators. Collectively, these strategies enable pathogens to take control of host transcriptional programs. This reprogramming underlies diverse virulence strategies, including activation of susceptibility genes for nutrient acquisition (Chen et al., 2010; Gupta et al., 2021), manipulation of host development to induce galls or tumors (Barash and Manulis-Sasson, 2007; Kay et al., 2007; Zuo et al., 2023), and suppression of immune responses (Moore et al., 2011; Khan et al., 2022).

Complementary to a recently published review on effectors targeting host TFs (Xiang et al., 2025), we highlight the complex mechanisms by which plant colonizing microbes manipulate host gene transcription. Understanding the function of effectors not only provides insights of infection mechanisms, but also expands our mechanistic understanding of eukaryotic gene expression and transcriptional regulation. Decoding the “TAL code” facilitated the identification of susceptible genes in the host and provided new strategies of disease control for *Xanthomonas* sp. Promoters containing effector binding elements (EBEs) can be engineered to drive resistance gene expression, creating a “trap” whereby pathogen TAL effectors specifically activate host defense. This strategy has been developed as a novel and targeted approach for controlling plant diseases (Schornack et al., 2013; Zeng et al., 2015; Shantharaj et al., 2023). Understanding the “TAL code” enabled the engineering of so-called TALENs (Transcription Activator-Like Effector Nucleases), DNA-binding proteins with customizable specificity that can be fused to different functional domains, such as nucleases or VP16 activation domains, to develop tools for genome editing or gene regulation (Tiley et al., 1992; Becker and Boch, 2021). A new functional group of transcriptional activator effectors is represented by Sts2. The further elucidation of the mechanism of Sts2 may lead to the finding of upstream TFs or DNA-binding protein controlling the expression of ZmSHR1, ZmGIF or ZmGRF, which are key regulators of maize leaf development (Zuo et al., 2023).

Effectors that function as transcription factors (TFs) or transcriptional co-regulators are particularly interesting because they actively manipulate host transcription. The identification of effectors that act as transcriptional activators raises several new questions: Are there additional transactivation effectors encoded by diverse microbes? What are their virulence functions, and how have they evolved and adapted during speciation? How can transcriptional activator effectors (TAEs) activate specific target genes despite lacking known DNA-binding domains? Is their specificity mediated through interactions with host proteins, or do they contain novel, as-yet-unidentified DNA-binding domains? First, previous effector candidates that exhibited autoactivation in yeast two-hybrid assays should be revisited, as they may represent potential TAEs that were previously overlooked. At present, TAE candidates can be predicted using a few publicly available bioinformatic tools, such as 9aaTAD (Piskacek et al., 2007) or TADA (Morffy et al., 2024), to identify potential transactivation domains (TADs). Nevertheless, these tools have limitations for the identification of fungal TAEs: 9aaTAD is based on experimentally characterized nine-amino-acid TADs from yeast and mammalian TFs (Piskacek et al., 2007), while TADA is trained on TADs identified from Arabidopsis TFs (Morffy et al., 2024). Both methods are limited for the prediction of many microbial effector proteins, which often lack known functional domains. Thus, a comprehensive experimental screen of TAEs from diverse pathogens, coupled with machine learning approaches to develop novel prediction tools, will be essential for advancing TAE research. In addition to TAEs, TF-like effectors may contain additional predicted DNA-binding domains. The application of AlphaFold to predict three-dimensional structures with potential novel DNA-binding capabilities will further expand our understanding of such effectors. Finally, it is likely that effectors can function as transcriptional co-repressors, inhibiting host gene transcription. However, the bioinformatics tools required to predict repression domains are not yet available. TPL/TPR-interacting effectors often contain an EAR motif. Identifying EAR motif-containing effectors and investigating whether they can function as transcriptional corepressors, interacting with certain transcription factors (TFs) and recruiting TPL/TPR to suppress the expression of their targets, would complement our knowledge of how pathogens manipulate host gene expression.
